# National trends and disparities in non-cancer mortality among older adults with oral cancer in the United States, 1999-2020

**DOI:** 10.3389/fonc.2026.1779819

**Published:** 2026-03-17

**Authors:** Yujie Jing, Yanbin Song, Hao Chen, Shuangyue Zhang, Meng Wu

**Affiliations:** Department of Stomatology, The Affiliated Huaian No.1 People's Hospital of Nanjing Medical University, Northern Jiangsu Institute of Clinical Medicine, Nanjing Medical University, Huaian, Jiangsu, China

**Keywords:** cardiovascular disease, non-cancer mortality, oral cancer, respiratory disease, temporal trends

## Abstract

**Background:**

As survival rates for oral cancer (OC) patients improve, non-cancer-related diseases, particularly cardiovascular disease (CVD) and respiratory disease (RD), pose significant threats to long-term health outcomes. Understanding shifts in non-cancer mortality is crucial for optimizing survivorship care. OC predominantly affects individuals of advanced age. This study aimed to analyze national trends in non-cancer mortality among older adults with OC and identify key factors contributing to these disparities.

**Methods:**

Mortality data from the CDC WONDER Multiple Cause of Death database were used to examine deaths among individuals aged ≥ 65 years from 1999 to 2020. Age-adjusted mortality rates (AAMRs) were computed, and temporal trends were assessed using Joinpoint regression. A decomposition analysis for 2015–2020 identified key factors driving recent increases in mortality rates.

**Results:**

A total of 7,460 non-cancer-related deaths were recorded, with 3,485 attributed to CVD (47%) and 1,472 to RD (20%). The overall AAMR was 0.837, with higher rates observed in males (1.197), White individuals (0.849), residents of the West (0.909), and those living in nonmetropolitan areas (0.874). Mortality declined from 1999 to 2014 but increased significantly thereafter (APC = 8.085), with the sharpest increases observed in females (APC = 11.358) and the Southern region (APC = 10.616). CVD mortality consistently exceeded RD mortality across all subgroups. Decomposition analysis indicated that population growth and rising age-specific mortality rates were the primary contributors to the observed increases in mortality.

**Conclusion:**

Non-cancer mortality, particularly from CVD and RD, is rising among older OC survivors, with notable demographic and geographic disparities. Integrating long-term cardiopulmonary management and enhancing chronic disease prevention efforts could reduce non-cancer mortality in this vulnerable population.

## Introduction

Oral cancer (OC) is a prevalent malignancy of the head and neck, marked by high incidence and mortality rates. Global cancer statistics report approximately 380,000 new cases and 190,000 deaths annually, with a 5-year survival rate of approximately 68% (1, 2). While advances in early detection and treatment have improved survival by nearly 20% over the past five decades (3), the proportion of deaths due to non-cancer causes has continued to rise as patient survival extends (4). Oral cancer predominantly affects individuals of advanced age, with incidence increasing markedly in older populations (5). Older adults are defined as individuals aged 65 years and older in epidemiological and clinical research (6). Older adults with OC are particularly vulnerable, as age-related physiological decline increases susceptibility to chronic comorbidities and mental health disorders (7). Among these, cardiovascular disease (CVD) and respiratory disease (RD) are the most common and have become leading contributors to non-cancer mortality (4, 8).

CVD remains the leading cause of death globally, encompassing ischemic heart disease, stroke, cardiac arrhythmias, and other cardiovascular conditions (9). In OC patients, CVD is the most frequent non-cancer cause of death (4). The proportion of cardiovascular mortality increases progressively with longer follow-up periods after an OC diagnosis, and this burden is expected to rise further as the follow-up duration extends (10).

RD is closely linked to cancer, with emerging evidence suggesting that RD not only represents an independent risk factor for malignancy but may also share common pathogenic mechanisms with cancer (11). Compared to the general population, OC patients exhibit significantly higher rates of chronic obstructive pulmonary disease, respiratory infections, and aspiration pneumonitis, particularly among older adults (12–14). Epidemiological studies further indicate that RD has become the second leading cause of non-cancer mortality among OC patients, especially among older individuals and long-term smokers (15).

Despite these observations, large-scale population-based epidemiological studies examining non-cancer mortality in OC—specifically deaths due to CVD and RD—are still scarce. The CDC WONDER (Wide-ranging Online Data for Epidemiologic Research) platform, developed by the U.S. Centers for Disease Control and Prevention, offers a comprehensive source of national mortality and demographic data. Using this resource, the current study aims to examine patterns and temporal trends in non-cancer-related mortality among older adults with OC, with a particular focus on deaths due to CVD and RD. The findings are intended to inform enhanced prevention, monitoring, and management strategies for this growing patient population.

## Methods

### Study design

This study utilized mortality data from the CDC WONDER platform, specifically from the Multiple Cause of Death database for the years 1999–2020. The analysis focused on long-term trends in non-cancer–related mortality among older adults with OC in the United States, with particular attention to deaths attributable to CVD and RD.

All data used in this study were sourced from publicly accessible, anonymized databases and did not include identifiable personal information; thus, the research was exempt from institutional ethical review.

### Data acquisition

Cohort selection followed a predefined extraction strategy from the CDC WONDER Multiple Cause of Death database. We first identified all decedents aged ≥65 years between 1999 and 2020. Among these, cases were included if oral cancer (ICD-10 codes C00–C06) was listed as either the underlying or contributing cause of death. Deaths attributed to non-cancer causes were then identified by excluding malignant neoplasms (C00–C97) as the underlying cause. Cardiovascular and respiratory disease deaths were further classified using ICD-10 codes I00–I99 and J00–J99, respectively.

Mortality records were stratified by sex, race, census region, and urbanization level. Sex was categorized as male or female, while race was classified as American Indian or Alaska Native, Black or African American, White, or Asian or Pacific Islander. The four census regions included the Northeast, Midwest, South, and West. Urbanization status was defined according to the 2013 National Center for Health Statistics Urban–Rural Classification Scheme for Counties, categorizing areas into metropolitan (large central metro, large fringe metro, medium metro, and small metro) and nonmetropolitan (micropolitan and noncore) groups (16). This stratification facilitated the evaluation of demographic and geographic differences in non-cancer mortality among OC patients.

### Statistical analysis

Descriptive analyses were performed to assess patterns in non-cancer mortality among older adults with OC, focusing particularly on CVD- and RD-related deaths. Mortality data were stratified by year, sex, race, census region, and urbanization level. For each subgroup, the number of deaths was recorded, and the age-adjusted mortality rate (AAMR) per 100,000 population was calculated using the direct standardization method, with the age distribution of the 2000 U.S. standard population applied. This approach allowed for valid comparisons across demographic groups and time periods.

Temporal trends in mortality were assessed using the Joinpoint Regression Program. Segmented log-linear regression and permutation testing were employed to identify statistically significant trend inflection points. For each identified segment, the Annual Percent Change (APC), Average Annual Percent Change (AAPC), and corresponding 95% confidence intervals (CIs) were calculated. A trend was considered statistically significant if the APC differed from zero, as determined by a two-tailed t-test with a significance threshold of P < 0.05.

To identify the drivers of the recent increase in mortality numbers, a decomposition analysis was conducted for the period 2015–2020, during which a marked upward trend in non-cancer mortality was observed. Changes in mortality counts were decomposed into three components: population growth, population aging, and changes in age-specific mortality rates (ASRs) (17). The results were presented using stacked bar plots, illustrating the relative contributions of each component to the overall changes in mortality.

## Results

### Overall non-cancer-related mortality and temporal trends

**Figure 1:**
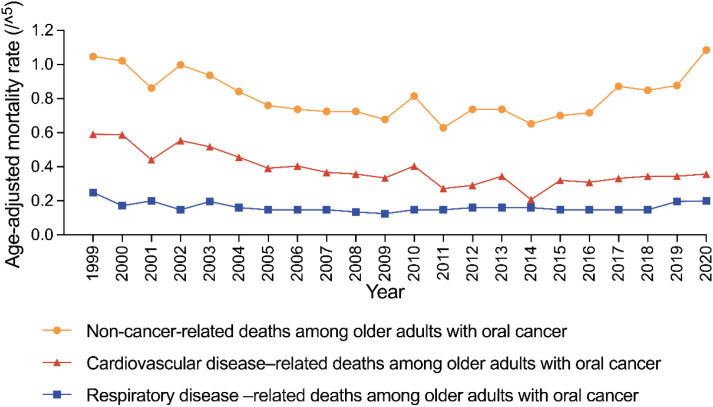
Trends in age-adjusted mortality rates for non-cancer, cardiovascular disease-related, and respiratory disease-related deaths among older adults with oral cancer in the United States, 1999–2020.

**Figure 2:**
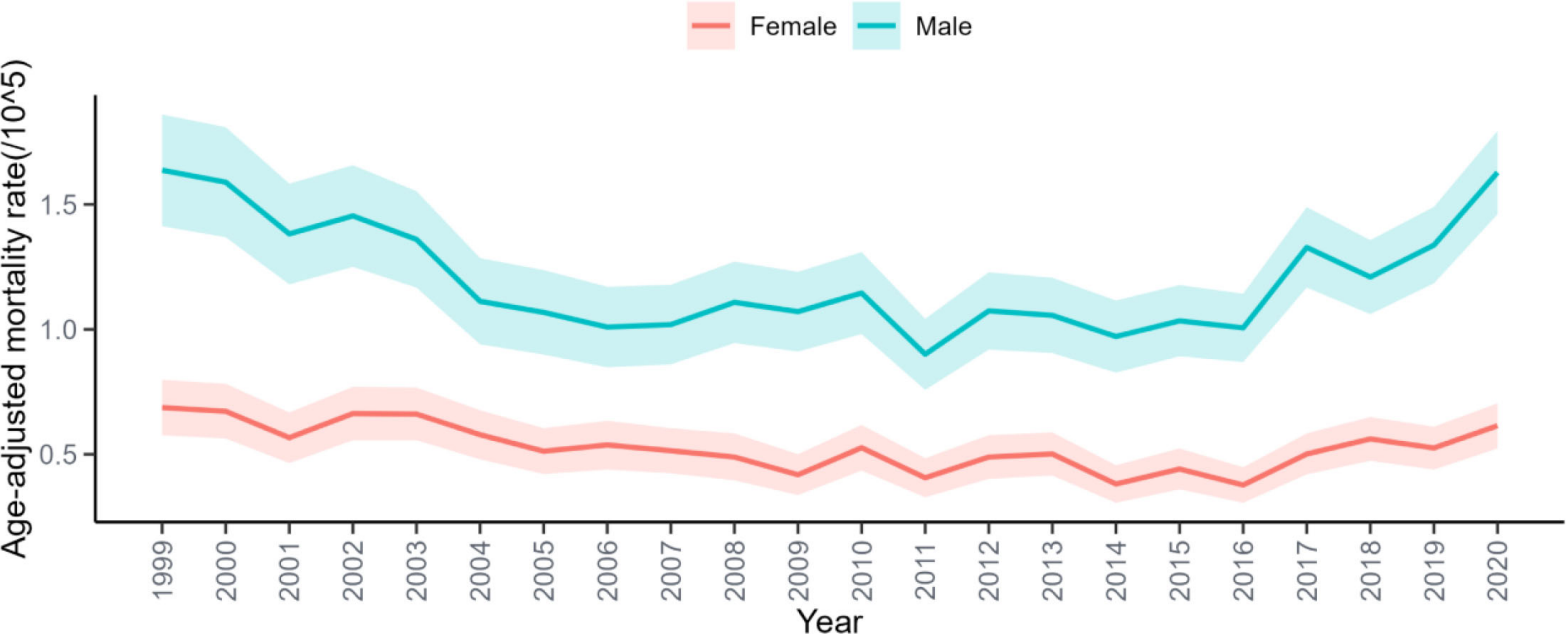
Trends in age-adjusted non-cancer mortality rates among older adults with oral cancer by sex, 1999–2020.

**Figure 3:**
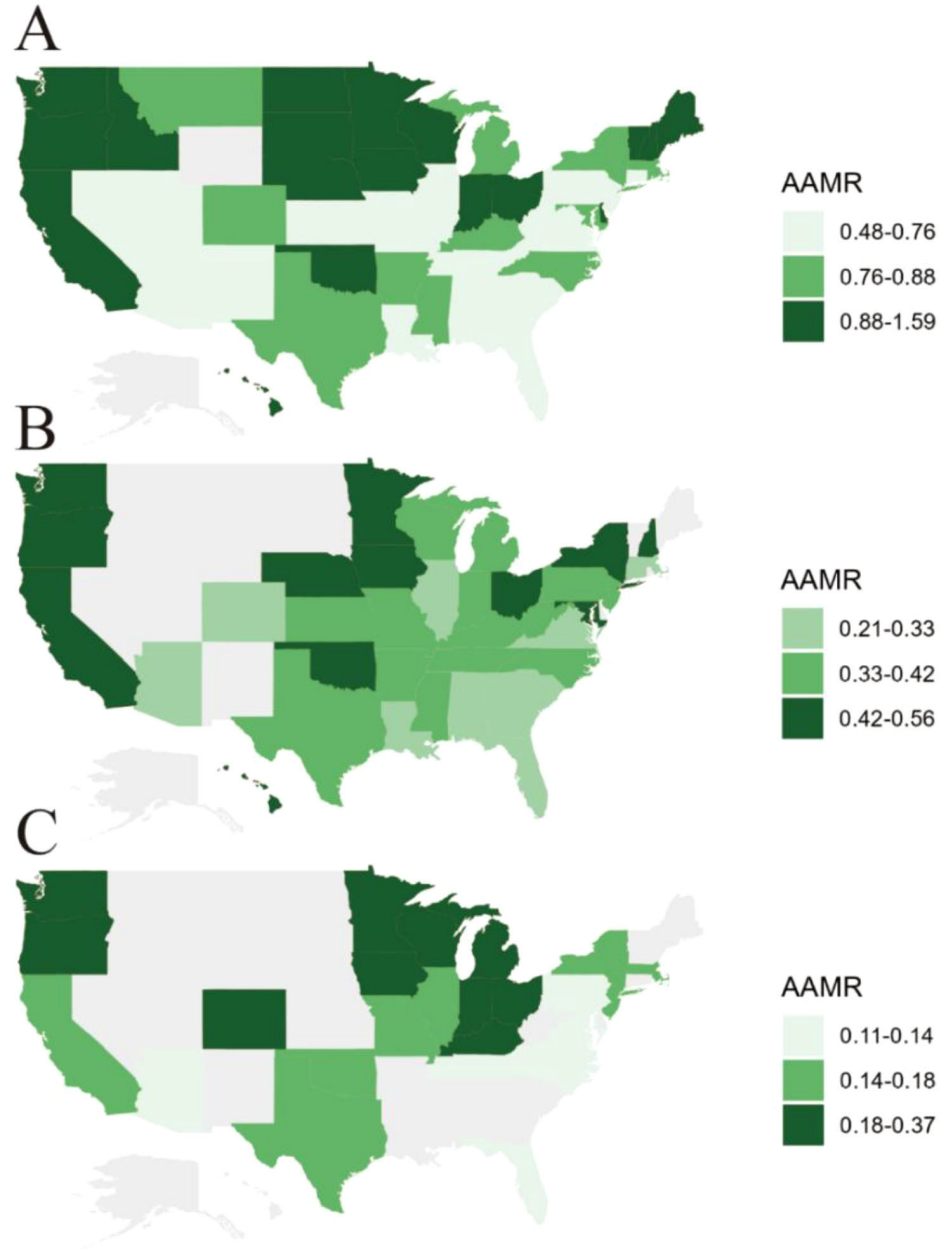
State-level age-adjusted mortality rates among older adults with oral cancer in the United States, 1999–2020. **(A)** Age-adjusted mortality rates (AAMRs) for non-cancer deaths among older adults with oral cancer across U.S. states. **(B)** AAMRs for cardiovascular disease-related deaths among older adults with oral cancer. **(C)** AAMRs for respiratory disease-related deaths among older adults with oral cancer.

From 1999 to 2020, a total of 7,460 older adults with OC died from non-cancer causes. As shown in **Table 1**, the overall AAMR was 0.837 (95% CI: 0.818–0.856), with males experiencing nearly double the mortality rate of females (1.197 vs. 0.501). **Figure 1** illustrates the temporal pattern, showing a decline in non-cancer mortality from 1999 to 2014, followed by a marked increase thereafter. Consistent trends by sex are presented in **Figure 2**, with males consistently exhibiting higher AAMRs across all years. Geographic variation was also notable, with the West showing the highest regional AAMR and the South the lowest. Vermont and Nebraska exhibited the highest state-level mortality rates. These spatial patterns are summarized in **Figure 3**. Differences between metropolitan and nonmetropolitan areas were modest, with slightly higher rates in nonmetropolitan populations (0.874 vs. 0.825) (**Table 1**).

**Table 1:** Baseline demographic characteristics and mortality rates of older adults with oral cancer who died from non-cancer causes in the United States, 1999–2020.

	All years		1999		2020	
Variable	Deaths/Population	AAMR per 100000 (95%CI)	Deaths/Population	AAMR per 100000 (95%CI)	Deaths/Population	AAMR per 100000 (95%CI)
Overall	7460/928476665	0.837 (0.818, 0.856)	364/34797841	1.047 (0.94, 1.155)	559/55659365	1.085 (0.994, 1.175)
Sex						
Female	2912/527632126	0.501 (0.483, 0.52)	148/20497330	0.687 (0.576, 0.798)	179/30837857	0.614 (0.523, 0.704)
Male	4548/400844539	1.197 (1.162, 1.232)	216/14300511	1.637 (1.413, 1.86)	380/24821508	1.628 (1.461, 1.794)
Race						
American Indian or Alaska Native	33/6051868	0.594 (0.406, 0.839)	–	–	–	–
Asian or Pacific Islander	142/35919615	0.454 (0.379, 0.528)	–	–	–	–
Black or African American	425/83274492	0.532 (0.481, 0.584)	22/2863945	0.812 (0.509, 1.229)	29/5481266	0.53 (0.352, 0.767)
White	6860/803230690	0.849 (0.829, 0.869)	340/30965588	1.095 (0.979, 1.212)	515/46873084	1.132 (1.034, 1.231)
Census Region						
Midwest	1834/207118111	0.872 (0.832, 0.912)	95/8247014	1.131 (0.915, 1.382)	131/11855295	1.12 (0.926, 1.314)
Northeast	1481/179714300	0.801 (0.761, 0.842)	70/7344915	0.924 (0.72, 1.168)	103/10049658	1.046 (0.843, 1.25)
South	2341/343442253	0.689 (0.661, 0.717)	114/12356583	0.971 (0.792, 1.149)	194/21205545	0.923 (0.792, 1.055)
West	1804/198202001	0.909 (0.867, 0.951)	85/6849329	1.249 (0.997, 1.544)	131/12548867	1.071 (0.885, 1.257)
Urbanization						
Metro	6012/760496648	0.825 (0.804, 0.846)	296/28137890	1.071 (0.949, 1.193)	467/46266874	1.085 (0.986, 1.184)
Nonmetro	1448/167978547	0.874 (0.829, 0.919)	68/6659951	0.973 (0.756, 1.234)	92/9391021	1.038 (0.836, 1.275)

### CVD- and RD-related mortality

Among all non-cancer deaths, CVD accounted for 47% (3,485 deaths) and RD for 20% (1,472 deaths). CVD-related mortality was higher among males, White individuals, residents of the Western region, residents of California, and nonmetropolitan populations. RD-related mortality was highest among White individuals, residents of the Midwest, residents of Minnesota, and nonmetropolitan areas. Across all demographic groups, the AAMR for CVD was consistently higher than that for RD (**Table 2**).

**Table 2:** Demographic characteristics of oral cancer decedents due to cardiovascular disease or respiratory disease in the United States, 1999–2020.

		Cardiovascular disease		Respiratory diseases	
Variable	Population	Deaths	AAMR per 100000 (95%CI)	Death	AAMR per 100000 (95%CI)
Overall	928476665	3485	0.357 (0.345, 0.369)	1472	0.148 (0.140, 0.155)
Sex					
Female	527632126	1349	0.257 (0.243, 0.271)	528	0.112 (0.102, 0.122)
Male	400844539	2136	0.552 (0.528, 0.576)	944	0.260 (0.243, 0.277)
Race					
Asian or Pacific Islander	35919615	72	0.245 (0.191, 0.309)	23	0.060 (0.034, 0.097)
Black or African American	83274492	239	0.272 (0.237, 0.307)	43	0.088 (0.063, 0.119)
White	803230690	3161	0.405 (0.390, 0.419)	1403	0.160 (0.152, 0.168)
Census Region					
Midwest	207118111	795	0.392 (0.365, 0.420)	414	0.196 (0.176, 0.215)
Northeast	179714300	753	0.405 (0.375, 0.434)	254	0.148 (0.129, 0.166)
South	343442253	1084	0.345 (0.324, 0.365)	443	0.148 (0.134, 0.162)
West	198202001	853	0.452 (0.422, 0.483)	361	0.172 (0.154, 0.190)
Urbanization					
Metro	760496648	2809	0.357 (0.344, 0.370)	1171	0.148 (0.139, 0.156)
Nonmetro	167978547	676	0.417 (0.385, 0.449)	301	0.160 (0.142, 0.178)

Both CVD- and RD-related AAMRs showed significant increases after 2014, reflecting the overall trend in non-cancer mortality (**Figure 1**).

### Temporal trends across subgroups

From 1999 to 2020, the AAMR for non-cancer mortality exhibited a modest long-term increase, driven primarily by sharp rises post-2014 (APC = 8.085; P = 0.006). Increases were observed in both sexes after 2016, with a more pronounced rise in females (APC = 11.358; P = 0.024) compared to males (APC = 10.860; P = 0.001). All census regions experienced recent increases, with the South showing the steepest growth (APC = 10.616; P = 0.023). Metropolitan areas showed a greater rate of increase than nonmetropolitan areas (**Table 3**).

**Table 3:** Annual percentage change and average annual percentage change in non-cancer mortality among patients with oral cancer in the United States.

Variable	Segment	Segment start	Segment end	APC (95% CI)	*P*-Value	AAPC (95% CI)	*P*-Value
Total	1	1999	2014	-2.869 (-7.002, -1.389)	0.005	0.142 (-1.102, 1.137)	0.653
	2	2014	2020	8.085 (2.206, 27.249)	0.006		
Sex							
Female	1	1999	2016	-2.869 (-6.151, -1.697)	0.008	-0.307 (-1.784, 0.660)	0.563
	2	2016	2020	11.358 (1.058, 30.607)	0.024		
Male	1	1999	2006	-6.523 (-14.959, -3.538)	0.018	-0.278 (-1.089, 0.534)	0.441
	2	2006	2016	0.014 (-3.334, 3.568)	0.993		
	3	2016	2020	10.86 (5.143, 22.782)	0.001		
Race							
White	1	1999	2020	-0.220 (-1.470, 1.158)	0.787	-0.220 (-1.47, 1.158)	0.787
Census Region							
Midwest	1	1999	2009	-4.255 (-7.417, -2.294)	<0.001	-0.284 (-1.005, 0.512)	0.466
	2	2009	2020	3.468 (1.883, 6.320)	<0.001		
Northeast	1	1999	2014	-2.307 (-9.148, -0.499)	0.023	0.326 (-1.164, 1.582)	0.571
	2	2014	2020	7.226 (0.971, 26.120)	0.020		
South	1	1999	2015	-3.188 (-10.593, -1.299)	0.014	-0.067 (-1.887, 1.307)	0.983
	2	2015	2020	10.616 (1.06, 35.728)	0.023		
west	1	1999	2011	-4.105 (-10.968, -1.864)	0.004	-1.012 (-2.194, 0.165)	0.094
	2	2011	2020	3.269 (0.212, 14.288)	0.037		
Urbanization							
Metro	1	1999	2014	-3.226 (-6.726, -1.724)	0.002	-0.007 (-1.228, 1.014)	0.893
	2	2014	2020	8.518 (2.709, 27.517)	0.003		
Nonmetro	1	1999	2009	-4.396 (-16.075, -1.139)	0.009	-0.264 (-1.561, 1.148)	0.702
	2	2009	2020	3.647 (1.035, 14.317)	0.011		

CVD-related mortality followed a similar upward trajectory after 2014, with significant increases noted among males, residents of the Southern region, and metropolitan populations. RD-related mortality also increased, though at a more moderate rate (**Table 4**).

**Table 4:** Annual percentage change and average annual percentage change in cardiovascular disease or respiratory disease mortality among patients with oral cancer in the United States.

Variable	Segment	Segment start	Segment end	APC (95% CI)	*P*-value	AAPC (95% CI)	*P*-Value
Cardiovascular disease mortality with oral cancer							
Total	1	1999	2014	-4.898 (-6.807, -3.722)	<0.001	-2.160 (-3.118, -1.257)	<0.001
	2	2014	2020	5.034 (0.349, 15.745)	0.036		
Sex							
Female	1	1999	2016	-5.319 (-11.368, -3.786)	0.025	-2.830 (-4.776, -1.440)	<0.001
	2	2016	2020	8.498 (-3.449, 37.417)	0.249		
Male	1	1999	2014	-4.697 (-6.820, -3.316)	<0.001	-1.322 (-2.321, -0.372)	0.005
	2	2014	2020	7.646 (2.557, 21.027)	0.003		
Race							
White	1	1999	2020	-2.322 (-3.524, -1.099)	<0.001	-2.322 (-3.524, -1.099)	<0.001
Census Region							
Midwest	1	1999	2010	-5.667 (-16.265, -3.029)	0.005	-2.232 (-3.715, -0.746)	0.003
	2	2010	2020	1.692 (-1.459, 17.042)	0.304		
South	1	1999	2015	-5.287 (-8.441, -3.843)	<0.001	-2.112 (-3.591, -0.436)	0.024
	2	2015	2020	8.781 (0.387, 33.027)	0.042		
Urbanization							
Metro	1	1999	2014	-5.146 (-6.732, -4.036)	<0.001	-2.236 (-3.103, -1.294)	<0.001
	2	2014	2020	5.436 (1.020, 14.928)	0.018		
Respiratory disease mortality with oral cancer							
Total	1	1999	2008	-5.293 (-15.698, -1.964)	0.004	-0.814 (-1.866, 0.438)	0.187
	2	2008	2020	2.684 (0.723, 9.428)	0.011		
Gender							
Male	1	1999	2009	-6.560 (-13.566, -3.182)	<0.001	-0.422 (-1.602, 0.963)	0.519
	2	2009	2020	5.508 (2.852, 11.159)	<0.001		
Race							
White	1	1999	2020	-0.177 (-1.488, 1.283)	0.872	-0.177 (-1.488, 1.283)	0.872
Urbanization							
Metro	1	1999	2002	-16.559 (-26.720, -7.946)	<0.001	-1.585 (-2.357, -0.514)	0.002
	2	2002	2014	-0.982 (-4.232, 1.253)	0.344		
	3	2014	2020	5.584 (2.247, 17.094)	0.004		

In the overall U.S. population aged ≥65 years, a significant increase in non-cancer mortality emerged during 2018–2020 (APC = 8.191; P = 0.002). In contrast, cardiovascular disease mortality showed a relatively stable trend (2012–2020: APC = −0.467; P = 0.098). Respiratory disease mortality exhibited a different trajectory, remaining largely unchanged before 2018 but showing a significant decline during 2018–2020 (APC = −4.478; P < 0.001) (**Supplementary Table 1**).

### Decomposition of population changes

**Figure 4:**
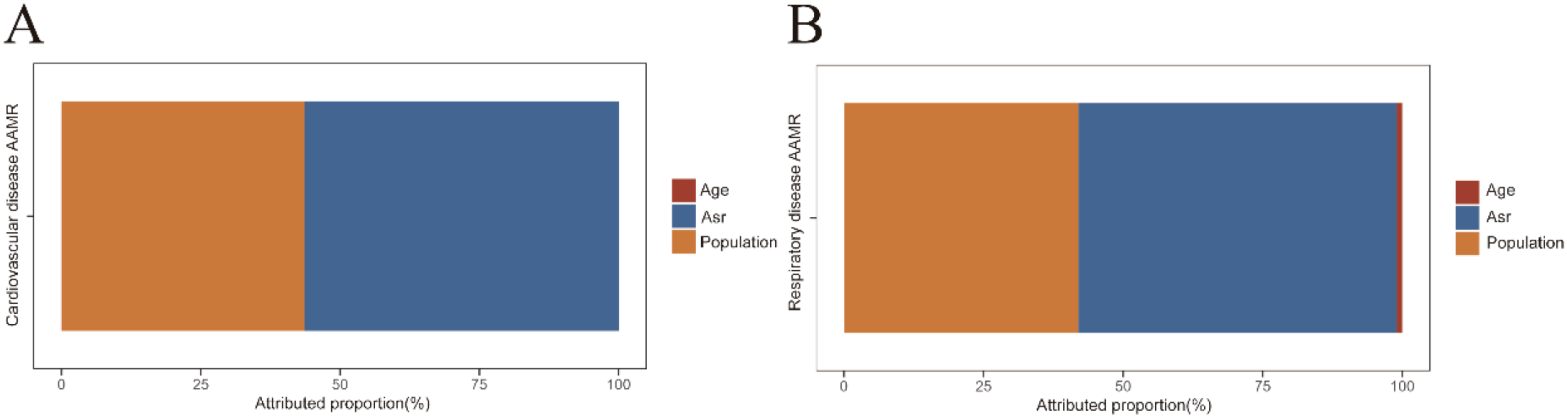
Decomposition of changes in mortality numbers among older adults with oral cancer, 2015–2020. **(A)** Relative contributions of population growth, population aging, and changes in age-specific mortality rates (ASRs) to the increase in cardiovascular disease-related deaths among older adults with oral cancer. **(B)** Relative contributions of population growth, population aging, and changes in age-specific mortality rates (ASRs) to the increase in respiratory disease-related deaths.

Decomposition analysis from 2015 to 2020 revealed that the increases in CVD- and RD-related mortality among older adults with OC were primarily driven by rising ASRs, with population growth serving as an additional contributing factor (**Figure 4**).

## Discussion

This nationwide study highlights a concerning rise in non-cancer mortality among older adults with oral cancer after 2014, particularly from cardiovascular and respiratory causes, underscoring emerging survivorship challenges in this population. These results are consistent with accumulating evidence highlighting non-cancer causes as a significant contributor to long-term mortality in head and neck cancer patients. Rose et al. demonstrated that nearly 28% of patients died from non-cancer causes within five years (18). This suggests that non-cancer mortality is becoming a critical aspect of survivorship, emphasizing the need to investigate the factors driving this trend.

Recent advances in OC diagnosis and management have significantly improved survival rates. High-resolution MRI has enhanced the accuracy of depth of invasion assessment, a key prognostic factor and an essential element in preoperative risk stratification (19). Pathologically, immunohistochemistry and molecular biomarkers have enabled more precise tumor characterization and better prognostic differentiation (20). Surgical techniques have evolved as well, with specimen-driven margin assessment and intraoperative imaging guidance improving margin adequacy and enhancing control of the primary tumor (21, 22). Additionally, postoperative adjuvant therapy has become a cornerstone of modern multimodal treatment, further contributing to higher cure rates (23).

This study identified CVD as the leading cause of non-cancer mortality and RD as the second most common cause, both of which have shown significant increases since 2014. Understanding the underlying mechanisms driving these trends is crucial. A combination of treatment-related, physiological, and behavioral factors likely contributes to the rising burden of cardiopulmonary deaths among older adults with OC.

CVD remains the leading non-cancer cause of death, with its AAMR increasing significantly in recent years. Several mechanisms may explain this trend. First, surgical treatment can directly lead to cardiovascular complications. Surgical trauma activates sympathetic pathways, elevates myocardial oxygen demand, and induces systemic inflammation, all of which increase the risk of perioperative cardiovascular events (24). Second, systemic therapies used in OC treatment often carry inherent vascular toxicity. For instance, 5-fluorouracil, a commonly used chemotherapeutic agent, is linked to cardiotoxicity rates ranging from 1% to 20%, manifesting as chest pain and, in severe cases, arrhythmia, myocardial infarction, or cardiac arrest (25). Furthermore, neck radiotherapy—commonly employed in curative and adjuvant settings (23)—has well-documented late cardiovascular effects. About 30% of patients develop carotid artery stenosis or cardiovascular events within 8 years of treatment, highlighting the long-term vascular consequences of radiation exposure (26). Beyond treatment-related toxicity, postoperative lifestyle changes may further elevate CVD risk. Older adults with OC often adopt high-calorie, easily digestible diets while reducing physical activity, which fosters obesity, insulin resistance, and dyslipidemia—factors that collectively increase long-term cardiovascular risk (27).

RD is the second leading contributor to non-cancer mortality, with its AAMR also showing a steady increase over time. Several factors may explain this rise. Cancer treatments can directly impair swallowing function, making aspiration pneumonia a major respiratory complication. Surgical interventions may alter oral and pharyngeal anatomy, leading to dysphagia, while radiotherapy induces fibrosis in swallowing-related musculature, further limiting mobility (12). Chemotherapy often causes mucositis, edema, and ulceration of the upper aerodigestive tract, which can hinder effective swallowing and airway protection (28). Multimodal therapy—including surgery, radiotherapy, and chemotherapy—poses the highest risk for aspiration pneumonia (12). Post-treatment functional limitations, along with taste changes and psychological distress, frequently result in reduced food intake and malnutrition. Malnutrition, in turn, compromises immune function and weakens respiratory mucosal defenses, increasing susceptibility to respiratory infections (29).

From 1999 to 2020, the AAMRs for CVD- and RD-related deaths were consistently higher in male older adults with OC than in females. Behaviorally, men tend to have higher rates of smoking and alcohol consumption, along with generally lower adherence to health-promoting behaviors (30). Biologically, estrogen provides cardioprotective benefits to women by modulating inflammation, improving lipid metabolism, and enhancing vascular relaxation (31). Additionally, emerging evidence suggests that men may experience stronger local inflammatory and pulmonary responses to radiotherapy (32).

Throughout the study period, older adults with OC in the Western United States consistently exhibited the highest CVD-related AAMRs. This pattern reflects both healthcare and environmental factors. From a healthcare perspective, studies indicate that coastal and metropolitan regions, including the West Coast, concentrate a higher number of NCI-designated head and neck radiotherapy centers, resulting in higher radiotherapy utilization rates (33). However, radiotherapy can cause delayed cardiovascular injury, with complications such as carotid artery stenosis often appearing 5 to 10 years after treatment (26). These delayed effects contribute to the persistently high AAMR in this region during long-term follow-up. The Western region also faces unique environmental challenges. Frequent wildfires and elevated levels of fine particulate matter (PM_2.5_) pollution exacerbate health risks. Between 1984 and 2015, the annual area burned by wildfires in the Western United States nearly doubled (34). Chronic exposure to PM_2.5_ can induce systemic inflammation, oxidative stress, and endothelial dysfunction, accelerating atherosclerosis and triggering cardiovascular events (35). These environmental stressors further increase the long-term mortality risk among older adults with OC in this region.

After 2015, the AAMR for CVD among older adults with OC rose most rapidly in the Southern region. This escalation can be attributed to several factors. Key risk factors such as hypertension, diabetes, and smoking are more prevalent in Southern states (36), and these have long been established as major contributors to cardiovascular mortality (36). Additionally, the region faces structural social disadvantages, including low income, low educational attainment, and limited health insurance coverage (37). These factors are strongly linked to elevated cardiovascular mortality and collectively contribute to the rapid increase in AAMR observed in the South.

Decomposition analysis further identified the factors driving the rise in non-cancer-related mortality among older adults with OC, particularly from CVD and RD. The analysis revealed that changes in ASR and population growth were the primary contributors, with the increase in ASR being the dominant factor. This finding is consistent with broader national trends in the United States over the past decade. Since 2010, the decline in cardiovascular ASR has significantly slowed (38), while the ASRs of major cardiovascular subtypes, including hypertensive heart disease and heart failure, have risen (39).

Given the observed rise in cardiopulmonary mortality, targeted survivorship strategies are urgently needed. Structured cardiovascular and respiratory risk assessment should be integrated into long-term follow-up for oral cancer survivors, particularly those with pre-existing comorbidities or treatment-related toxicities. Multidisciplinary survivorship clinics, involving oncologists, cardiologists, pulmonologists, and primary care providers, may facilitate early identification of high-risk individuals and timely intervention. Furthermore, population-level prevention strategies - including smoking cessation, hypertension control, metabolic risk management, and air quality monitoring - remain essential components of comprehensive survivorship care.

This study has several limitations. First, all analyses were based on death certificate data from the CDC WONDER system, which lacks detailed clinical information such as tumor stage, treatment modalities, comorbidity profiles, and behavioral risk factors. Smoking, a major shared risk factor for oral cancer and cardiopulmonary diseases, may therefore represent an important unmeasured confounder, as individual-level tobacco exposure data were unavailable. Residual confounding related to cumulative lifetime smoking exposure cannot be excluded. Second, cause-of-death misclassification is an inherent limitation of death certificate–based data. Errors in death certificates are common and can extend to ICD−10 coding, potentially affecting mortality statistics and epidemiological inferences at the population level (40). Studies comparing death certificates with cancer registry records have demonstrated variability in the accuracy of cause-of-death coding, particularly when certification is completed by non-specialist physicians, indicating potential misclassification in cancer mortality data (41). Therefore, the potential for misclassification should be considered when interpreting the mortality trends observed in this study. Third, the dataset does not include diagnosis dates or follow-up duration, preventing an assessment of time since diagnosis and whether mortality trends vary across early, mid-, and late survivorship stages. Fourth, since our analysis focused on individuals aged ≥ 65 years, heterogeneity within older age subgroups could not be explored. Fifth, county- and state-level differences in healthcare access, environmental exposures, and socioeconomic conditions could not be incorporated, limiting the interpretation of geographic disparities. Finally, although decomposition analysis helped identify contributors to mortality changes from 2015 to 2020, the method does not capture all potential determinants, and the ecological nature of the analysis precludes causal inference.

## Conclusion

In this national analysis, non-cancer mortality among older adults with OC, particularly deaths from CVD and RD, showed a notable increase after 2014. Significant demographic and geographic disparities were observed, with males, White individuals, and residents of the West experiencing a higher burden. These findings highlight the growing impact of cardiopulmonary diseases on OC survivorship and underscore the need for enhanced long-term management and chronic disease prevention in this vulnerable population.

## Data Availability

Publicly available datasets were analyzed in this study. This data can be found here: The data used in this study are publicly available from the CDC WONDER, accessible at https://wonder.cdc.gov.
