# Supplementary material for: National trends and disparities in non-cancer mortality among older adults with oral cancer in the United States, 1999-2020
Source: Front Oncol. 2026 Mar 17;16:1779819. doi: 10.3389/fonc.2026.1779819 (PMC13035714; doi:10.3389/fonc.2026.1779819)
Supplement: Supplementary file 1 [file Table1.docx]

**Supplementary Table 1 Annual percentage change and average annual percentage change in non-cancer, cardiovascular disease or respiratory disease mortality in the United States**

| **Variable** | **Segment** | **Segment Start** | **Segment End** | **APC (95% CI)** | ***P*-value** | **AAPC (95% CI)** | ***P*-Value** |
| --- | --- | --- | --- | --- | --- | --- | --- |
| **Non-cancer mortality** | | | | | | | |
| Total | 1 | 1999 | 2009 | -1.9(-4.623, 1.012) | 0.059 | -0.442(-0.855, -0.14) | 0.025 |
|  | 2 | 2009 | 2018 | -0.65(-2.765, 1.028) | 0.31 |  |  |
|  | 3 | 2018 | 2020 | 8.191(2.572, 11.073) | 0.002 |  |  |
| **Cardiovascular disease mortality** | | | | | | | |
| Total | 1 | 1999 | 2003 | -2.978(-3.548, -1.69) | 0.002 | -2.495(-2.589, -2.363) | <0.001 |
|  | 2 | 2003 | 2007 | -5.141(-6.114, -4.365) | <0.001 |  |  |
|  | 3 | 2007 | 2012 | -3.172(-3.943, -1.069) | 0.014 |  |  |
|  | 4 | 2012 | 2020 | -0.467(-0.802, 0.228) | 0.098 |  |  |
| **Respiratory disease mortality** | | | | | | | |
| Total | 1 | 1999 | 2018 | -1.168(-1.99, 3.273) | 0.112 | -1.488(-1.851, -0.876) | <0.001 |
|  | 2 | 2018 | 2020 | -4.478(-8.35, -0.956) | <0.001 |  |  |
